# Phosphorylation Status of 72 kDa MMP-2 Determines Its Structure and Activity in Response to Peroxynitrite

**DOI:** 10.1371/journal.pone.0071794

**Published:** 2013-08-27

**Authors:** Anna Laura Jacob-Ferreira, Marcia Yuri Kondo, Pravas Kumar Baral, Michael N. G. James, Andrew Holt, Xiaohu Fan, Richard Schulz

**Affiliations:** 1 Department of Pediatrics, Cardiovascular Research Centre, Mazankowski Alberta Heart Institute, University of Alberta, Edmonton, Alberta, Canada; 2 Department of Pharmacology, Cardiovascular Research Centre, Mazankowski Alberta Heart Institute, University of Alberta, Edmonton, Alberta, Canada; 3 Department of Biochemistry, Cardiovascular Research Centre, Mazankowski Alberta Heart Institute, University of Alberta, Edmonton, Alberta, Canada; Aligarh Muslim University, India

## Abstract

Matrix metalloproteinase-2 (MMP-2) is a key intra- and extra-cellular protease which contributes to several oxidative stress related pathologies. A molecular understanding of 72 kDa MMP-2 activity, directly mediated by S-glutathiolation of its cysteine residues in the presence of peroxynitrite (ONOO^−^) and by phosphorylation of its serine and threonine residues, is essential to develop new generation inhibitors of intracellular MMP-2. Within its propeptide and collagen binding domains there is an interesting juxtaposition of predicted phosphorylation sites with nearby cysteine residues which form disulfide bonds. However, the combined effect of these two post-translational modifications on MMP-2 activity has not been studied. The activity of human recombinant 72 kDa MMP-2 (hrMMP-2) following *in vitro* treatments was measured by troponin I proteolysis assay and a kinetic activity assay using a fluorogenic peptide substrate. ONOO^−^ treatment in the presence of 30 µM glutathione resulted in concentration-dependent changes in MMP-2 activity, with 0.1–1 µM increasing up to twofold and 100 µM attenuating its activity. Dephosphorylation of MMP-2 with alkaline phosphatase markedly increased its activity by sevenfold, either with or without ONOO^−^. Dephosphorylation of MMP-2 also affected the conformational structure of the enzyme as revealed by circular dichroism studies, suggesting an increase in the proportion of α-helices and a decrease in β-strands compared to the phosphorylated form of MMP-2. These results suggest that ONOO^−^ activation (at low µM) and inactivation (at high µM) of 72 kDa MMP-2, in the presence or absence of glutathione, is also influenced by its phosphorylation status. These insights into the role of post-translational modifications in the structure and activity of 72 kDa MMP-2 will aid in the development of inhibitors specifically targeting intracellular MMP-2.

## Introduction

Posttranslational modifications such as phosphorylation, methylation, acylation, and hydroxylation can occur at several amino acid residues after protein synthesis [1]. Phosphorylation may alter the biological activity of an enzyme, determine a protein's subcellular targeting, affect interactions with binding partners, as well as label proteins for proteolysis [2], [3]. Addition of negatively charged phosphates to a protein's serine, threonine or tyrosine residues may change its characteristics, particularly its conformation [4], [5]. Phosphorylation is reversible and may also act as a molecular switch for enzyme activity [6].

Proteins may also undergo oxidative modification, both physiological and pathological, as a natural consequence of aerobic life, and in the pathological response to increased oxidative stress [7]. Oxidative modification of proteins ranges from the facile oxidation of cysteine residues to covalent crosslinking with other proteins and the formation of protein adducts with lipid, carbohydrate, or nucleic acid radicals [8].

Matrix metalloproteinase-2 (MMP-2) was widely considered to be a secreted, zinc-dependent endopeptidase which proteolyses extracellular matrix and non-matrix proteins in both physiological and pathological processes [9], [10], [11]. However, several studies have shown that MMP-2 can also cleave specific intracellular targets [12], [13] including the sarcomeric protein troponin I [14], thereby contributing to the acute contractile dysfunction seen following ischemia-reperfusion and other types of enhanced oxidative stress injury to the heart. Despite having a signal sequence associated with secreted proteins, canonical MMP-2 is inefficiently targeted to the endoplasmic reticulum for secretion, resulting in its prominent cytosolic localization [15]. In addition, human cardiomyocytes also express an MMP-2 splice variant lacking the signal sequence which restricts it to the cytosol [15]. These findings explain the intracellular localization of MMP-2 and why several intracellular matrix substrates, including troponin I, have been found for it [12], [13], [16].

MMP-2 is synthesized as a 72 kDa zymogen and can be activated in the extracellular space by proteolytic removal of its autoinhibitory propeptide to render an active 64 kDa MMP-2 [9]. Alternatively, peroxynitrite (ONOO^−^), the prooxidant reaction product of nitric oxide and superoxide [17], activates 72 kDa MMP-2 via a non-proteolytic mechanism involving the S-glutathiolation of a cysteine sulfhydryl moiety in its propeptide. This occurs at low (0.3–1.0 µM) concentrations of ONOO^−^ in the presence of intracellular glutathione (GSH). In contrast, concentrations of ONOO^−^ higher than 100 µM cause profound protein oxidation and loss of MMP-2 catalytic activity as a result of irreversible oxidation of critical cysteine and other residues [18].

MMP-2 activity is also regulated by its phosphorylation status. MMP-2 from human sarcoma cells contains 29 predicted phosphorylation sites. Five of these were confirmed by mass spectrometry and occur on residues with side chains accessible at the surface of the protein. Phosphorylation of 64 kDa MMP-2 *in vitro* with protein kinase C markedly diminishes, whereas MMP-2 dephosphorylation by alkaline phosphatase enhances the activity of MMP-2 [19]. The phosphorylation status of MMP-2 in the heart affects its activity as the phosphatase inhibitor okadaic acid enhanced MMP-2 phosphorylation and reduced its activity, thereby protecting hearts from ischemia-reperfusion induced contractile dysfunction [20].

Despite knowledge that MMP-2 is unique amongst all 23 known human MMPs in that it is also localized within cells [15], is activated by ONOO^−^ mediated S-glutathiolation [18] and it is also a phosphoprotein [19], no one has examined the combined effect of both ONOO^−^ and its phosphorylation status on its enzymatic activity. Furthermore, it is unknown whether changes in MMP-2 phosphorylation result in changes to its conformational structure. We therefore, using 72 kDa MMP-2 as a prototypical intracellular MMP, examined changes in its conformational structure relating to its phosphorylation status and changes in the activity of both phosphorylated and dephosphorylated forms following its S-glutathiolation with ONOO^−^. Since the influence of both S-glutathiolation and phosphorylation on MMP-2 activity is a realistic scenario within cells during conditions such as oxidative stress, this work will contribute towards the rational design of inhibitors targeting intracellular MMP-2.

## Methods

### Materials

Unless otherwise specified, chemical reagents were obtained from Sigma-Aldrich (Oakville, ON) or Fisher Scientific (Ottawa, ON). Human recombinant 72 kDa MMP-2, purified from TIMP-2^−/−^ mouse fibroblasts [21] stably over-expressing hrMMP-2, was a kind gift from Chris Overall (University of British Columbia, Vancouver, Canada). Human recombinant troponin (TnI) in 50 mM Tris pH 7.6 containing 0.5 M NaCl, 0.1 mM dithiothreitol and 0.1% (w/v) NaN_3_ was a gift from James Potter (University of Miami, Miami FL, USA). The following were purchased from the sources indicated: alkaline phosphatase from bovine intestinal mucosa (Sigma-Aldrich, St. Louis MO, USA), protein-A Sepharose bead suspension (BioVision, Mountain View CA, USA), mouse monoclonal IgG_1_ and mouse monoclonal glutathione antibodies (Abcam, Cambridge MA, USA), mouse monoclonal anti-human MMP-2 antibody (Millipore, Temecula CA, USA), secondary horseradish peroxidase-conjugated antibody (Transduction Laboratories, Mississauga, ON), ECL Plus (Amersham, Buckinghamshire, UK); OmniMMP® fluorogenic peptide substrate (Enzo Life Sciences, Plymouth Meeting PA, USA); Phos-tag™ acrylamide (NARD Institute Ltd., Amagasaki, Japan); Precision Plus protein dual color molecular weight standards and Coomassie Brilliant Blue R-250 (Bio-Rad, Hercules CA, USA).

### Preparation of peroxynitrite

Both ONOO^−^ and decomposed ONOO^−^ (DPN) were prepared by the method described by Villa et al. [22]. An ice-cold aqueous solution of NaNO_2_ (2 M) and a solution containing nitric acid (11.1 M) and H_2_O_2_ (8.2 M) were filled into two separate 10 ml syringes connected with a Y-piece tubing. The syringe contents were simultaneously discharged into a rapidly stirring solution of ice-cold NaOH (4.2 M). Excess H_2_O_2_ was removed by treating the mixture with 2 g of granular Mn(IV)O_2_ for 5 min and the solution was filtered through Whatman #54 filter paper. The concentration of ONOO^−^ was determined by UV spectroscopy (ε^302^ = 1.67 mM^−1^ cm^−1^). The final concentration of ONOO^−^ achieved in this process varied between 130 and 200 mM. Decomposed ONOO^−^ (DPN) was prepared in the similar way, except that the solutions of NaNO_2_ and H_2_O_2_ in nitric acid were not captured in the NaOH solution. After 5 min, at which time ONOO^−^ was completely decomposed, NaOH was then added. This solution was also treated with Mn(IV)O_2_ to remove excess H_2_O_2_ and filtered. The absence of ONOO^−^ in this solution was verified by UV spectroscopy. The stock solutions were aliquoted and stored at −80°C for up to 4 weeks.

### Prediction of phosphorylation sites within human MMP-2

The primary sequence of human MMP-2 (UniProt ID: P08253) was obtained from the Swiss-Prot protein database (http://us.expasy.org/sprot/). NetPhos 2.0 (http://www.cbs.dtu.dk/services/NetPhos/), based on a neural network method, predicts serine, threonine, and tyrosine phosphorylation sites in eukaryotic proteins [23]. The three dimensional representation of MMP-2 showing predicted phosphorylation sites was prepared using the crystal structure available in the protein data bank (PDB) accession code: 1CK7.

### Dephosphorylation of 72 kDa MMP-2 with alkaline phosphatase and determining its phosphorylation status

Since native MMP-2 expressed in mammalian cells is partially phosphorylated [19], hr72 kDa MMP-2 (14 nM) was incubated in 50 mM Tris buffer pH 7.6 with or without alkaline phosphatase (100 U/µg target protein) for 20 min at 37°C. To confirm changes in its phosphorylation status, MMP-2 (1.5 µg/well for SDS-PAGE and 0.5 µg/well for Western blot) was analyzed using 10% SDS-PAGE under reducing conditions. Separate gels used for the separation of phosphorylated proteins contained in addition 100 µM Phos-tag acrylamide and 200 µM MnCl_2_. Following electrophoresis, samples were stained with Coomassie blue. With MnCl_2_ as a binding agent, Phos-tag was immobilized in the acrylamide gel. As proteins move through the gel, phosphorylated proteins were reversibly captured by Phos-tag, resulting in a mobility shift. Proteins with a greater degree of phosphorylation are delayed running through the gel and thus appear to have a higher molecular weight than their less phosphorylated forms.

### Circular dichroism (CD) studies of hrMMP-2

To determine changes in the secondary structure of MMP-2 using CD we required a concentration range of 0.1–0.7 mg/ml of pure protein. For this purpose, we expressed and purified hrMMP-2 produced in mouse TIMP-2^−/−^ fibroblasts [21] (a kind gift from Chris Overall, University of British Columbia, Vancouver, Canada). Briefly, a lentiviral construct EX-hMMP2-Lv129 vector containing human MMP-2 cDNA and Halotag at its C-terminus was obtained from Genecopoeia (Rockville, MD). Lentiviral vectors were produced by co-transfecting 293T cells with the expression vector and three packaging plasmids. The fibroblasts were transduced with the vector and selected by supplementing 2 µg/ml puromycin in full culture media to generate a stable expressing cell line. The cells were cultivated in T175 flasks for 3–4 days until 80% confluence and conditioned medium was collected, centrifuged to remove cells and debris and stored at −80°C until bulk purification. Purification of conditioned medium containing hrMMP-2 was performed at 4°C with gelatin sepharose chromatography (gelatin sepharose 4B, GE Healthcare Buckinghamshire, UK) in MES (2-(N-morpholino)ethanesulfonic acid) buffer A (50 mM MES, 5 mM CaCl_2_, 0.1 M NaCl, 0.025% sodium azide, pH 6.0) and eluted with MES buffer B (MES buffer A +10% DMSO). Eluted fractions containing MMP-2 were pooled, confirmed using SDS polyacrylamide gel electrophoresis and dialyzed into 50 mM Tris buffer, pH 7.6. Concentration of the protein was determined by bicinchoninic acid protein assay. The fraction intended for dephosphorylation was incubated with alkaline phosphatase (100 U/µg target protein) for 20 min at 37°C, the control (native) fraction was also incubated for 20 min at 37°C but without alkaline phosphatase. In order to remove both MMP-9 co-expressed by the fibroblasts and the alkaline phosphatase used for dephosphorylation, both fractions were then subjected to a second step of purification at 4°C with HaloLink™ resin. hrMMP-2 bound to HaloLink resin was cleaved by HaloTEV protease and eluted with buffer (50 mM HEPES, 150 mM NaCl, 1 mM dithiothreitol, 0.005% octylphenoxypolyethoxyethanol). Pooled samples were dialysed to 50 mM Tris–HCl pH 7.5 buffer and concentrated to 0.2 mg/ml. Purity of both native and dephosphorylated MMP-2 was determined as seen in a Coomassie blue stained SDS-PAGE gel and confirmed as a single band of 72 kDa (Fig. S1).

Circular dichroism (CD) studies were performed on a DSM-17 CD spectrophotometer (OLIS Instruments, Bogart GA, USA) fitted with a Peltier system for controlling cell temperature. Native hrMMP-2 and that treated with alkaline phosphatase were diluted in 50 mM Tris–HCl pH 7.5 buffer and spectra of 0.2 mg/ml protein solutions were collected at 30°C in the far UV range (190–260 nm) using a 0.2 mm path length quartz cell and analyzed by means of OLIS Spectral Works software (version 4.8.11); data were normalized in terms of mean residual ellipticity. The baseline control was obtained using the buffer vehicle without added MMP-2. Estimates of secondary structure composition were obtained by deconvolution using CDPro [24], and CDNN software version 2.1 (Copyright Gerald Böhm, Institut für Biotechnologie, Martin-Luther Universität Halle-Wittenberg).

### Treatment of 72 kDa MMP-2 with ONOO^−^ and GSH

Native hrMMP-2 or that treated with alkaline phosphatase (dephosphorylated MMP-2) were further treated with ONOO^−^ or DPN in the presence or absence of GSH (30 µM) in 50 mM Tris buffer pH 7.6, 37°C. Due to its short half-life at physiological pH [17], ONOO^−^ (0.1–100 µM) was added as three additions at 5 min intervals.

### Immunoprecipitation study to determine MMP-2 glutathiolation

To investigate whether the treatment of 72 kDa MMP-2 with ONOO^−^ (0.3 µM) and/or GSH (30 µM) resulted in its activation due to S-glutathiolation, native or dephosphorylated MMP-2 (4 ng/µl), which had been treated with ONOO^−^ with or without GSH, was incubated with or without dithiothreitol (1.0 mM) in Tris buffer (50 mM Tris-HCl, 3.1 mM sucrose, pH 8.0) for 10 min at 30°C. Dithiothreitol reverses S-glutathiolation and was used as a control [18]. Samples were incubated with either mouse monoclonal IgG_1_ (negative control, 1∶100 dilution) or mouse monoclonal glutathione antibody (1∶250 dilution) at 4°C overnight in 50 mM Tris-HCl, 3.1 mM sucrose, pH 8.0. Protein A-Sepharose bead suspension (1∶10 v/v) was added and further incubated under agitation at 4°C overnight. After centrifugation (12,000 *g*, 30 s), the supernatant was discarded and the pellet washed three times in 50 mM Tris-HCl, 3.1 mM sucrose, pH 8.0 at 4°C. The final pellet was suspended in 10 µl sample buffer and boiled at 95°C for 5 min and centrifuged. The supernatant was used for SDS-PAGE electrophoresis followed by Western blotting for MMP-2.

Gels were then electroblotted onto polyvinylidene difluoride membranes in Towbin buffer (20% methanol, 25 mM Tris, 192 mM glycine and 0.05% w/v sodium dodecyl sulphate). Molecular weight standards were also loaded onto gels to assist in identifying proteins of interest. Membranes were blocked with 5% w/v skim milk powder in TTBS buffer (0.001% v/v Tween-20, 2 M Tris pH 7.6, 0.1 M NaCl) for 1 h at room temperature and then probed overnight with a mouse anti-human MMP-2 antibody (1∶1000 dilution). Blots were then probed with mouse secondary horseradish peroxidase-conjugated antibody for 1 h at room temperature and incubated with ECL Plus for 5 min before being exposed to film. Band intensities were quantified using ImageJ software (National Institutes of Health, USA).

### Mass spectrometry

hr72 kDa MMP-2 (native or dephosphorylated, 0.1 µM) was treated or not with 0.3 µM ONOO^−^ in the presence of 30 µM GSH in 50 mM Tris pH 7.6. Mass spectra (MALDI-TOF MS) were obtained after in-solution trypsin digestion, under conditions as described [18].

### OmniMMP hydrolysis

The hydrolysis of 25 µM OmniMMP fluorogenic peptide by 10 nM native (phosphorylated) or dephosphorylated 72 kDa MMP-2 treated, in the presence or absence of 30 µM GSH, with ONOO^−^ (0–100 µM) or DPN was measured at 37°C every 30 s for 1 h, as described [19]. This assay was done on four separate occasions, with two different sample sets of MMP-2 per treatment group for each experiment, resulting in a final N = 7–8/group.

### Troponin I proteolysis

Native or dephosphorylated 72 kDa MMP-2 (14 nM), were treated with ONOO^−^ (0–0.3 µM) or DPN (±30 µM GSH) and incubated for 120 min at 37°C with 2 µM TnI in 50 mM Tris pH 7.6, 10 mM CaCl_2_, 0.05% Brij, 10 µM ZnSO_4_. Samples were diluted in reducing Laemmli buffer (containing 6% (v/v) 2-mercaptoethanol) [25], boiled for 3 min, run on a 10% SDS-PAGE gel, and stained with Coomassie blue. The appearance of lower molecular weight bands below TnI (25 kDa) indicated TnI proteolysis. This assay was done on four separate occasions, with one or two different sample sets of MMP-2 per treatment group for each experiment, resulting in a final N = 4–7/group.

### Gelatin zymography

To confirm that changes in MMP-2 activity were a result of non-proteolytic post-translational modifications we performed gelatin zymography. Native or dephosphorylated 72 kDa MMP-2, treated with ONOO^−^ and or GSH, was diluted in non-reducing Laemmli buffer (0.5 M Tris pH 6.8, 30% glycerol, 10% (w/v) SDS, 0.012% (w/v) bromophenol blue), loaded (0.5 ng/well) onto an 8% polyacrylamide gel copolymerized with 2 mg/ml gelatin, and electrophoresed for 90 min (125 V). The gel was rinsed in 2.5% (v/v) Triton X-100 (3×20 min), incubated for 16 h in 50 mM Tris buffer containing 5 mM CaCl_2_ and 150 mM NaCl, stained with 0.05% Coomassie Brilliant Blue G in 10% acetic acid containing 25% (v/v) methanol, and distained in 8% (v/v) acetic acid containing 4% methanol. Gelatinolytic activities were detected as transparent bands against the background of Coomassie blue-stained gelatin. To quantify the activities of the detected enzymes, zymograms were imaged using a GS-800 Calibrated Densitometer (Bio-Rad). The intensities of the separated bands were analyzed using ImageJ version 1.36b software (National Institutes of Health) and reported as such.

### Statistical analysis

Data are expressed as means ± SEM. Statistical analyses were performed using one-way analysis of variance (ANOVA) and Fisher's LSD *post hoc* test. p<0.05 was considered statistically significant.

## Results

### Phosphorylation status of MMP-2

**Figure 1:**
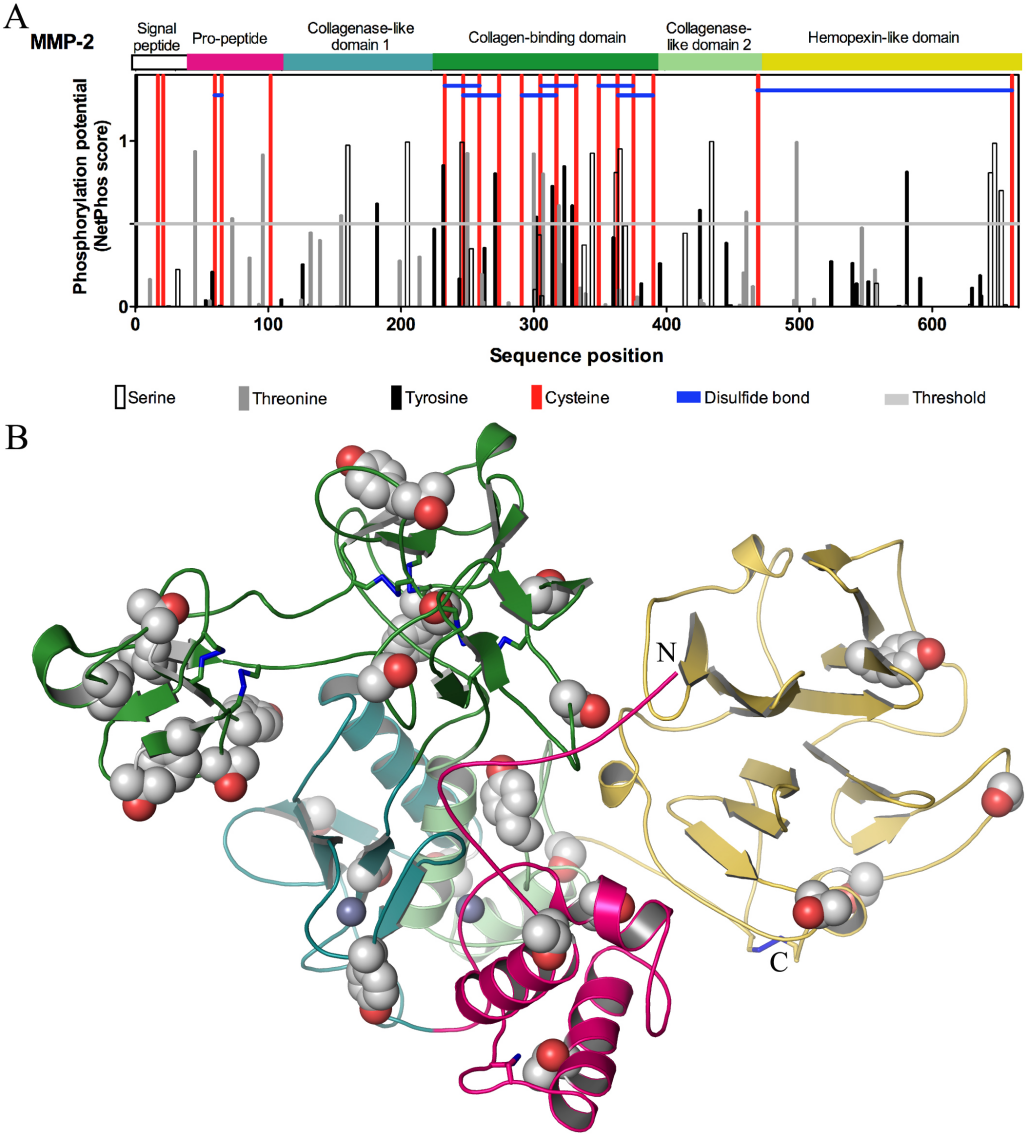
Potential phosphorylation sites of MMP-2. (A) Domain structure, NetPhos predicted phosphorylation sites, cysteine residues and disulphide bonds of human MMP-2. Vertical white, grey and black lines that extend above the demarcated horizontal threshold indicate putative amino acid phosphorylation sites, whether serine, threonine, or tyrosine, respectively. Red vertical lines indicate all cysteine residues present in the MMP-2 sequence, and blue horizontal lines represent the disulphide bonds of human MMP-2. (B) Crystal structure of MMP-2 (PDB ID: 1CK7) is shown in cartoon representation. The pro-peptide (hot pink), collagenase-like domain 1 (deep teal), collagenase-binding domain (forest green), collagenase-like domain 2 (pale green) and hemopexin-like domain (yellow) are shown in the indicated colours. Zn^2+^ ions are indicated as purple spheres. Potential phosphorylation sites (serine, threonine and tyrosine) are shown in Corey-Pauling-Koltun (CPK)/space filling representation, with carbon and oxygen atoms of the side chains in grey and red, respectively.

**Figure 2:**
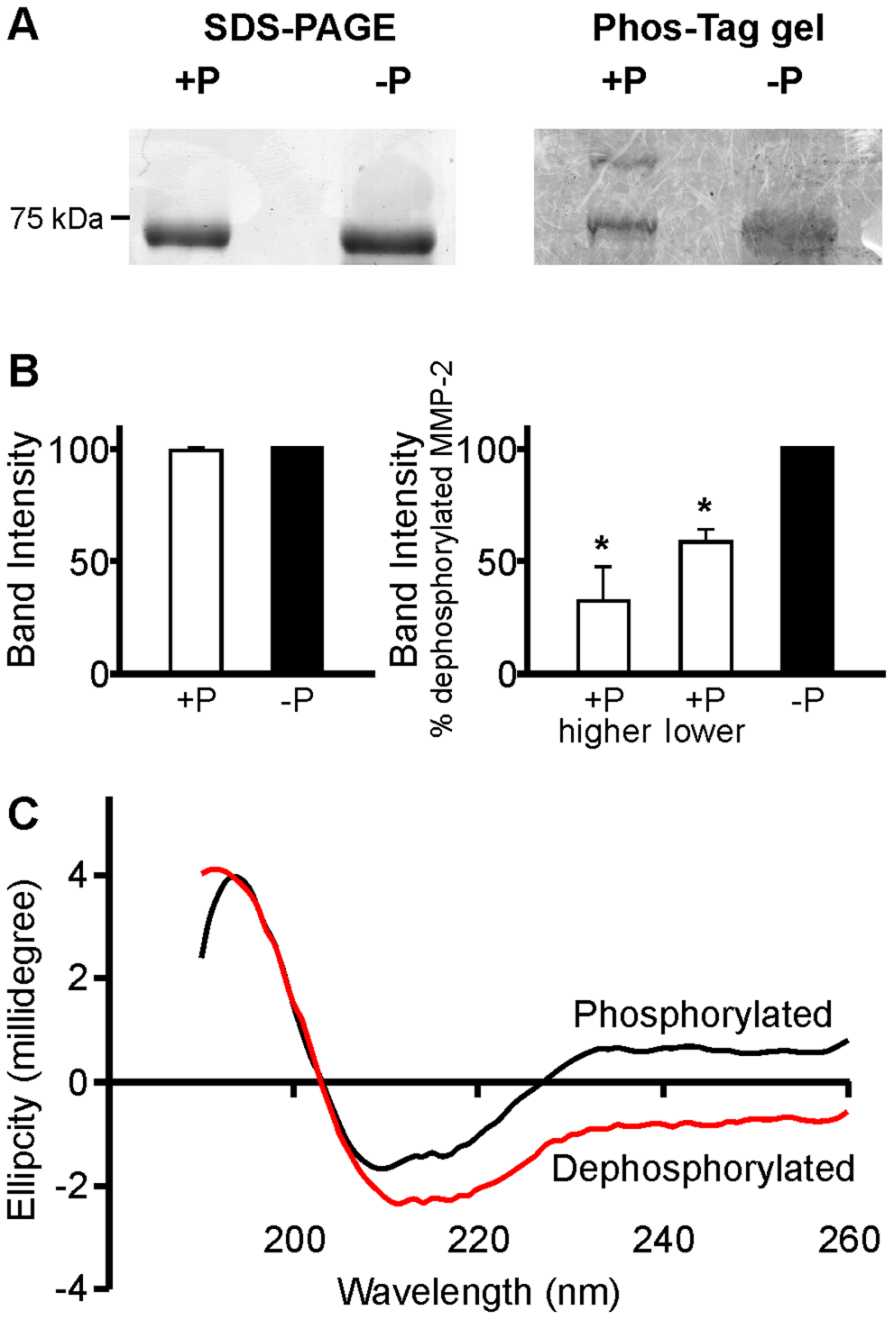
Phosphorylation status of 72 kDa MMP-2 and changes in its secondary structure following dephosphorylation. (A) In representative Coomassie blue stained SDS-PAGE gels, both native (phosphorylated, +P) and dephosphorylated (−P) 72 kDa MMP-2 run with the same pattern, showing bands only at 72 kDa (left). However, when run on the Phos-tag acrylamide gel, a more slowly migrating band also appeared, indicating that a portion of MMP-2 is phosphorylated. Phos-tag gel revealed at least two different phosphorylation states of 72 kDa MMP-2 and confirmed its dephosphorylation after treatment with alkaline phosphatase (right, N = 4). (B) The ratio of band intensity of total MMP-2 measured by SDS-PAGE (left), as well as ratio of higher to lower band intensities measured in the Phos-tag gel (right, mean ± SEM, N = 4), comparing phosphorylated to dephosphorylated samples, is demonstrated in the bar graphs below. (C) CD spectra of hrMMP-2. Far UV range CD spectrum of native (phosphorylated, black line) and dephosphorylated (red line).

We previously showed that hrMMP-2 and MMP-2 secreted from HT1080 cells are phosphorylated [19]. Analysis of phosphorylation sites within human MMP-2 using NetPhos predicted that there are 29 potential phosphorylation sites; it is also shown that six of the eight disulfide bonds of MMP-2 are found in the collagen-binding domain and one is found in the propeptide domain (Fig. 1A). The distribution of the predicted phosphorylation sites in the structure of MMP-2 is represented in the three dimensional crystal structure in Fig. 1B. Fourteen of these predicted phosphorylation sites are within the collagen-binding domain, an essential element for substrate binding, and three of these were confirmed (at threonine 250, tyrosine 271 and serine 365) by proteomic analysis [19]. There are four predicted sites in the collagenase-like domain 1 and three in the collagenase-like domain 2. The COOH-terminal hemopexin-like domain that is believed to contribute to substrate specificity [26] presents five predicted sites. Interestingly, three predicted sites are within the propeptide domain, a crucial region for enzyme activation. Phos-tag acrylamide gel analysis of the hr72 kDa MMP-2 utilized in this study shows that at least two different phosphorylation states of MMP-2 are present (Fig. 2A). This protease is partially phosphorylated in its native form, and treatment with alkaline phosphatase for 20 min at 37°C resulted in its significant dephosphorylation. Coomassie blue stained gels showed that samples of both native (phosphorylated) and dephosphorylated MMP-2 run at the same molecular weight of approximately 72 kDa in SDS-PAGE (Fig. 2A, left). However, when analyzing an identical aliquot of MMP-2 using the Phos-Tag gel, a phosphorylated form of MMP-2 appeared as a more slowly migrating band, which disappeared upon treatment with alkaline phosphatase (Fig. 2A, right). The percentage of intensities (Fig. 2B) of the lowest band (dephosphorylated MMP-2) was 58.4±6.1% comparing native (phosphorylated) to dephosphorylated samples, while the percentage of the higher band to the dephosphorylated band was 32.3±15.1% (N = 4). Phosphorylated MMP-2 may have separated into several bands when using Phos-Tag gel due to different possible phosphorylation moieties of MMP-2 [19]. However, we did not observe more than one additional band given the limitations of protein loading and detection of proteins using Coomassie blue staining of Phos-Tag gels.

We performed circular dichroism (CD) studies to evaluate whether phosphorylation status could affect the secondary structure of MMP-2 (Fig. 2C). Deconvolution of CD data and analysis of CD spectra using several different algorithms for both native and dephosphorylated hrMMP-2 samples indicated an average increase of 50% in α-helices length and an average decrease of 17% in β-strands length when MMP-2 is dephosphorylated, implying that the removal of phosphate groups from MMP-2 by alkaline phosphatase affected its secondary conformation.

### S-glutathiolation of MMP-2 after ONOO^-^ treatment

**Figure 3:**
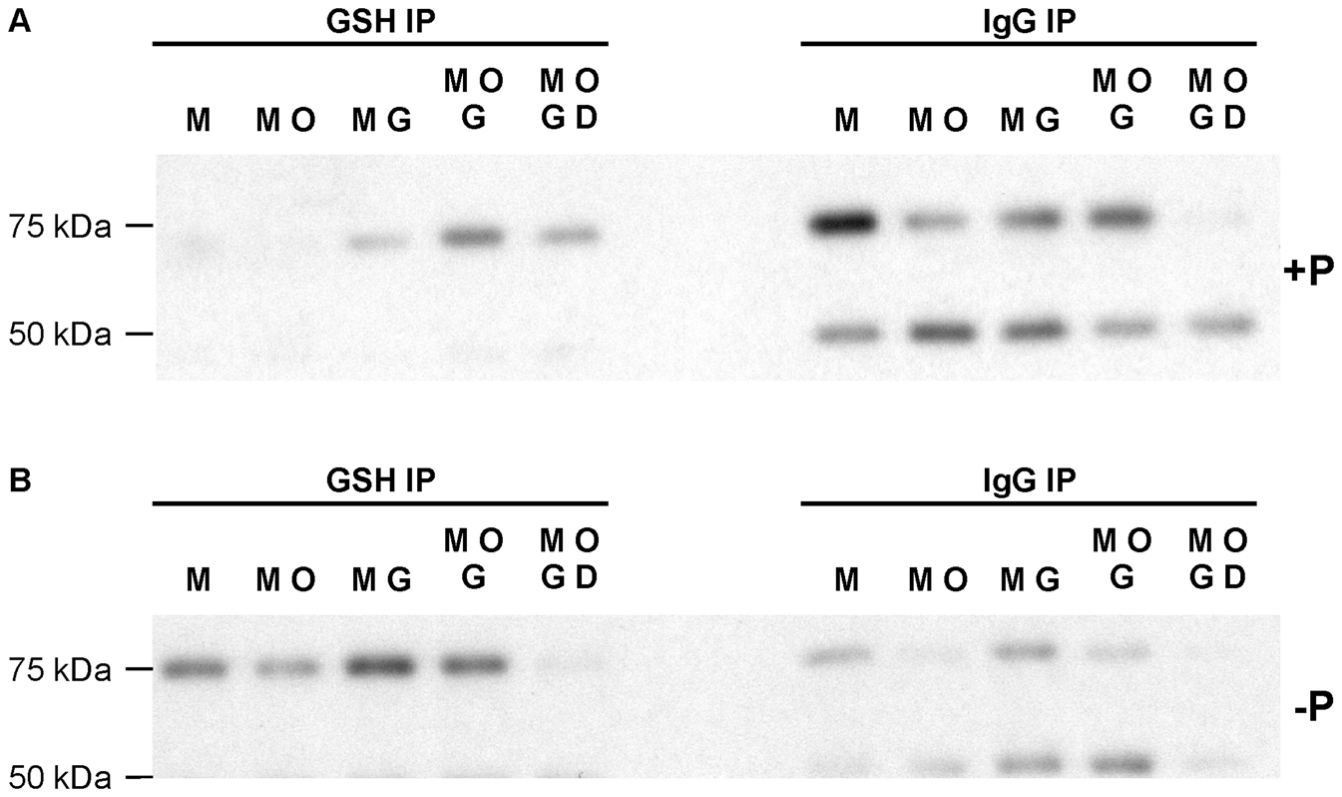
S-glutathiolation of phosphorylated and dephosphorylated 72 kDa MMP-2 after ONOO^−^/GSH treatment. S-glutathiolation of phosphorylated (A) or dephosphorylated (B) MMP-2, treated with 0.3 µM ONOO^−^ in the presence or absence of 30 µM GSH was determined by immunoprecipitation with glutathione (GSH) antibody, or IgG as a control, followed by Western blot for MMP-2. M, MMP-2; O, 0.3 µM ONOO^−^; G, 30 µM GSH; D, 1 mM dithiothreitol.

We then determined the glutathiolation status of both native and dephosphorylated 72 kDa MMP-2 upon immunoprecipitation with anti-GSH. This revealed that treatment of native MMP-2 with 0.3 µM ONOO^−^ and 30 µM GSH resulted in a significant glutathiolation of the enzyme (Fig. 3A). Interestingly, when MMP-2 was dephosphorylated, a more intense band appears in the Western blot for MMP-2 after immunoprecipitation with anti-GSH (Fig. 3B). Comparing these results with the immunoprecipitation control with unrelated IgG, the presence of MMP-2 (≈75 kDa) in a different pattern of bands from immunoprecipitation with anti-GSH can be seen. For the dephosphorylated sample, the band of MMP-2 after immunoprecipitation with anti-GSH may be due to a change in MMP-2 conformation resulting in enhanced epitope accessibility following removal of phosphate groups. The reducing agent dithiothreitol reversed the S-glutathiolation of both phosphorylated and dephosphorylated MMP-2. Mass spectrometry analysis of phosphorylated and dephosphorylated MMP-2, treated or not with GSH and ONOO^−^, showed S-glutathiolation of MMP-2 in two positions: Cys 60 and Cys102 (Table 1). Cys 60 is identified as the one forming the disulfide bridge (Cys 60 to Cys 65) present in the loop of the propeptide domain of MMP-2 that accommodates the site that is cleaved by MMP-14 upon its proteolytic activation [27], [28] and Cys 102 was confirmed as the predicted glutahiolated residue that can be activated by ONOO^−^ [18], [28]. It is noteworthy that dephosphorylation does not prevent S-glutathiolation, and as previously shown [19] it may also modulate the activity of MMP-2 activated by proteolysis (to 64 kDa MMP-2), suggesting that conformational changes by dephosphorylation are not dependent upon the propeptide domain uncovering the catalytic site.

**Table 1:** Mass spectrometry data obtained for native and dephosphorylated MMP-2 treated with 0.3 µM ONOO^−^ and 30 µM GSH showing modifications in Cys residues.

72 kDa MMP-2	coverage (%)	# unique peptides	glutathiolation
**+P**	32.58	12	ND
**+P, G+O**	35.91	14	C60, C102
**−P**	21.36	10	ND
**−P, G+O**	24.85	11	C60, C102

+P = native, −P = dephosphorylated, G+O = glutathione and ONOO^−^ treated, ND = not detected.

### Proteolysis of fluorogenic MMP substrate by ONOO^−^ treated MMP-2

**Figure 4:**
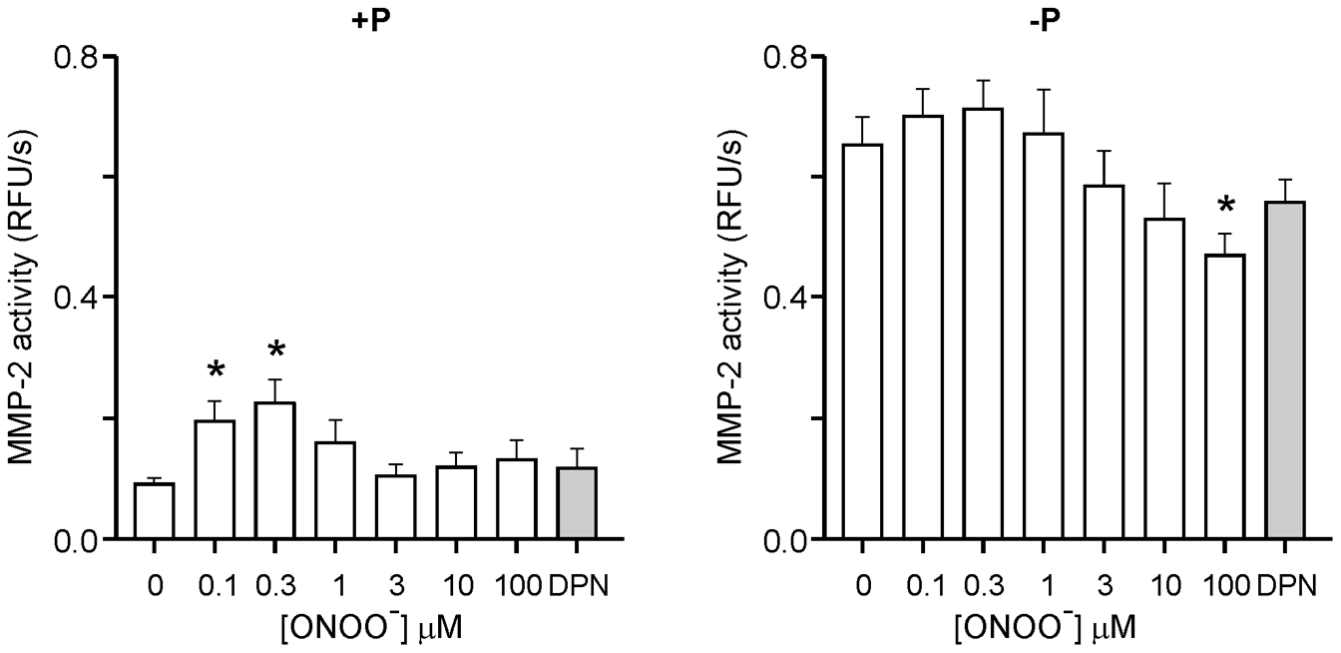
Effect of MMP-2 phosphorylation status in regard to activity changes following ONOO^−^ treatment using Omni MMP as a substrate. Proteolysis of fluorogenic Omni MMP substrate by ONOO^−^-treated, native (+P, left panel) or dephosphorylated (−P, right panel) 72 kDa MMP-2, in the presence of 30 µM GSH. Mean ± SEM, N = 7–8/group. *p<0.05 versus 0 µM ONOO^−^. DPN, decomposed peroxynitrite.

We investigated the effects of MMP-2 phosphorylation status, and further treatment with different concentrations of ONOO^−^, on MMP-2 activity by measuring the kinetics of hydrolysis of OmniMMP. Native MMP-2 treated with increasing concentrations of ONOO^−^ (in the presence of 30 µM GSH) caused a twofold increase in catalytic activity which peaked at 0.3 µM ONOO^−^ (Fig. 4, left panel, p<0.05). The increase in MMP-2 activity was lost at ONOO^−^ concentrations ≥1 µM. Decomposed ONOO^−^, in contrast, did not increase MMP-2 activity.

As previously shown [19] dephosphorylation of 72 kDa MMP-2 alone significantly increased its activity by sevenfold (Fig. 4, 0.09±0.01 vs. 0.65±0.05 RFU/s, p<0.0001). ONOO^−^ treatment of dephosphorylated MMP-2 resulted in a modest (but statistically non-significant) concentration-dependent increase in MMP-2 activity which also peaked at 0.3 µM ONOO^−^. In contrast, at higher concentrations (≥3 µM), ONOO^−^ caused a concentration-dependent reduction in MMP-2 activity which was maximal at 100 µM (Fig. 4, right panel, p<0.05). Thus, dephosphorylation of MMP-2 on its own resulted in a markedly greater increase in MMP-2 activity compared with the effect of ONOO^−^ on either dephosphorylated or native (phosphorylated) MMP-2.

To determine if the activation of hr72 kDa MMP-2 was promoted by loss of its propeptide domain or via non-proteolytic post-translational modifications, gelatin zymography was performed (Fig. S2). ONOO^−^ treated phosphorylated MMP-2 showed a 1.6 fold increase in activity and dephosphorylation caused a 3.5 fold increase in MMP-2 activity. Of note both ONOO^−^ and dephosphorylation treatments increased MMP-2 activity without proteolytic cleavage of the enzyme.

ONOO^−^ (0.1–100 µM) was unable to increase MMP-2 activity in the absence of GSH (N = 7–8/group, data not shown), consistent with ONOO^−^ activation being via its S-glutathiolation [18]. However, the marked increase in MMP-2 activity following dephosphorylation was maintained (0.07±0.01 vs. 0.64±0.07 RFU/s, p<0.0001, N = 7–8/group, data not shown).

### Troponin I degradation by ONOO^−^-treated native and dephosphorylated MMP-2

**Figure 5:**
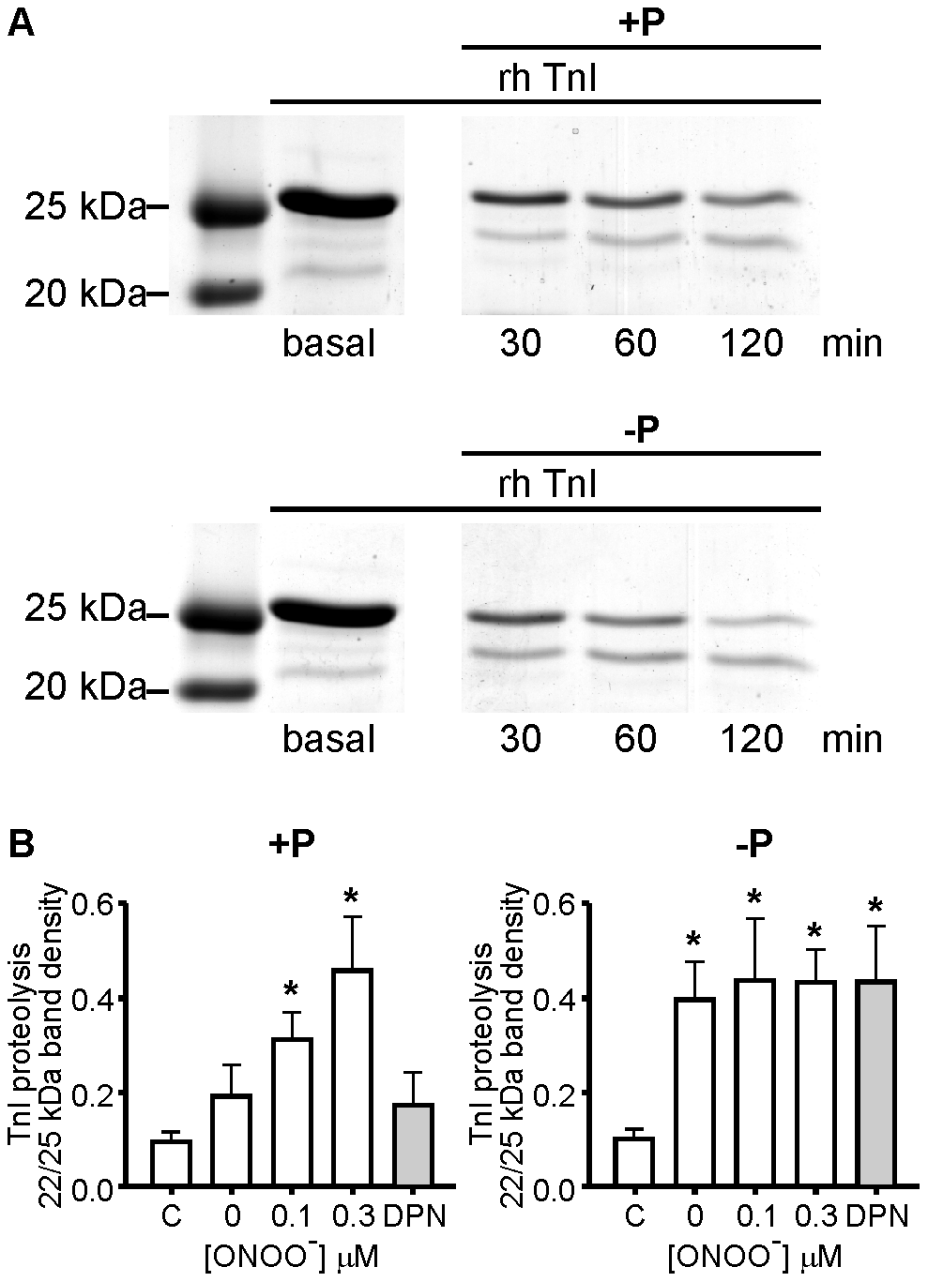
Effect of MMP-2 phosphorylation status in regard to activity changes following ONOO^-^ treatment using troponin I (TnI) as a known intracellular substrate. (A) Representative time-dependent troponin I (TnI) hydrolysis by 0.3 µM ONOO^−^ - treated, native (upper panel) or dephosphorylated (lower panel) 72 kDa MMP-2, in the presence of 30 µM GSH, following different incubation times (30, 60, or 120 min) at 37°C with TnI. Representative Coomassie blue stained SDS-PAGE gels. (B) Quantitative analysis of TnI hydrolysis by native (left) or dephosphorylated (right) 72 kDa MMP-2, treated with different concentrations of ONOO^−^ (0–0.3 µM) or DPN, in the presence of 30 µM GSH. Incubation for 120 min at 37°C. Mean ± SEM, N = 4–7/group. * p<0.05 compared with control (C, TnI alone). DPN, decomposed peroxynitrite.

We next analyzed the effects of both phosphorylation and glutathiolation on MMP-2 activity using troponin I (TnI). TnI is an endogenous substrate for MMP-2 in the cardiac sarcomere, to which it localizes in the thin myofilaments [14]. ONOO^−^-treated native MMP-2 proteolysed TnI, as evidenced by the appearance of a lower molecular weight product of approximately 23 kDa and a decrease in the intensity of intact TnI. TnI proteolysis occurred in a time-dependent manner, regardless of whether native or dephosphorylated MMP-2 was used (Fig. 5A). Hydrolysis of TnI by ONOO^−^-treated, native MMP-2 peaked at 0.3 µM ONOO^−^ (Fig. 5B, left). In contrast, the higher basal activity of dephosphorylated MMP-2 obliterated any additional effect of ONOO^−^ on its activity (Fig. 5B, right).

## Discussion

This study demonstrates for the first time that the phosphorylation status of 72 kDa MMP-2 modulates its activation by ONOO^−^ with GSH and also that changes in its secondary structure occur as a result of phosphorylation. Post-translational modifications of 72 kDa MMP-2 by oxidative stress have been postulated to be a key event leading to its intracellular activation in various pathological conditions [12], [13], [18].

We previously showed that ONOO^−^ can activate 72 kDa MMP-2, with increased activity at low concentrations of ONOO^−^, and a loss of enzymatic activity at higher concentrations of ONOO^−^, by a modification of the cysteine residue in the autoinhibitory domain of MMP-2 that is involved in enzyme activation [18]. We did not, however, determine the phosphorylation status of MMP-2 in that study, although some proportion of hrMMP-2 is already phosphorylated [19]. Similar concentration-dependent modulation of 72 kDa MMP-2 activity by ONOO^−^ in the presence of GSH was observed in this study. MMP-2 is a phosphoprotein and its dephosphorylation significantly increases its enzymatic activity [19]. A similar enhancement in the catalytic cleavage of substrates associated with MMP dephosphorylation was observed in this study, when comparing native and dephosphorylated MMP-2.

Although phosphorylation is a well known post-translational modification capable of dramatic alterations of protein secondary structure [29], the effect of phosphorylation/dephosphorylation on MMP-2 structure had not been determined. This study is the first to show structural analysis of MMP-2 which demonstrates that the secondary structure of dephosphorylated MMP-2 differs from native (phosphorylated) MMP-2 with a predicted increase in α-helices and decrease in β-strands upon dephosphorylation of the protein. These data suggest that the conformational structure of 72 kDa MMP-2 can directly affect its activity. However in order to define the precise structural alterations that occur due to phosphorylation/dephosphorylation and how they affect the catalytic mechanism of MMP-2, more detailed structural studies using nuclear magnetic resonance and x-ray crystallography are necessary.

Using both artificial (OmniMMP) and endogenous (TnI) substrates to test its activity, we observed that dephosphorylated MMP-2 showed higher activity in comparison with S-glutathiolated, native MMP-2, especially when OmniMMP was used as a substrate. The effect of enzyme dephosphorylation was only modestly enhanced by further treatment with ONOO^−^. One explanation for this observation is that dephosphorylation of MMP-2 may change its conformation in order to facilitate the exposure of the catalytic domain, without requiring the opening of the cysteine switch in the propeptide domain, as occurs when phosphorylated MMP-2 is glutathiolated. This hypothesis is supported by the fact that even when MMP-2 is activated by proteolysis (to 64 kDa MMP-2), its enzymatic activity may be further markedly enhanced by its dephosphorylation [19].

Human 72 kDa MMP-2 contains 29 potential phosphorylation sites (Figure 1). One can observe that the majority of the predicted phosphorylation sites are within the collagen-binding domain, which is an essential element for substrate binding [19]. It is also in this region that six of the 8 disulfide bonds of MMP-2 are located. Therefore, we suggest that the dephosphorylation of MMP-2 may change its conformation in a way that activates the enzyme even in the presence of its propeptide domain. The precise phosphorylation sites important for regulating MMP-2 activity, and through which activation (upon dephosphorylation) occurs via conformational changes, still need study.

Oxidants can induce protein phosphorylation [30], [31], as well as reduce phosphatase activity [32]. This may be the reason why the activity of dephosphorylated MMP-2 is decreased with a high concentration of ONOO^−^ (100 µM). Peroxynitrite at this concentration could have also decreased alkaline phosphatase activity, reducing the dephosphorylation rate of MMP-2, and consequently its activity.

S-glutathiolation occurred in both native and dephosphorylated MMP-2 with low concentrations of ONOO^−^. However, one critical point for S-glutathiolation is the ratio between ONOO^−^ and GSH (1∶100 in this study), as GSH can also act as an ONOO^−^scavenger. Another observation is that these experiments were performed in a non-physiological buffer (50 mM Tris buffer pH 7.6). In the presence of physiological buffers containing sodium bicarbonate and carbon dioxide, ONOO^−^ reacts and forms nitrosoperoxocarboxylate (ONOOCO_2_^−^) which is also an important prooxidant species [33] and could enhance ONOO^−^ effects in the activation of MMP-2 by S-glutathiolation.

In summary, we have shown that the ONOO^−^-induced changes in 72 kDa MMP-2 activity, an ubiquitous MMP expressed in nearly all cell types having proteolytic actions both inside and outside of cells [12], [13], [16], is further regulated by its phosphorylation status. This extends the hierarchy of the complex means of regulating this protease, beyond transcription and regulation by tissue inhibitors of metalloproteinases. Therefore, changes in MMP-2 phosphorylation status and cysteine oxidation may modify its conformational structure which results in changes in MMP-2 activity. This may be relevant to pathophysiological conditions associated with increased oxidative stress and intracellular activation of MMP-2, and for the development of inhibitors which precisely target its activated forms.

## Supporting Information

Figure S1 **Purity of hrMMP-2.** A) 10% Coomassie blue stained SDS-PAGE gel showing purified native (+P) or dephosphorylated (−P) hr 72 kDa MMP-2 used for CD studies. Expression and purification of hrMMP-2 were done as described in Methods.
(TIF)

Figure S2 **MMP-2 activity measured by gelatin zymography.** Representative gelatin zymogram for 0–0.3 µM ONOO^−^-treated, native (+P) or dephosphorylated (−P) 72 kDa hrMMP-2, in the presence of 30 µM GSH, following 16 h of incubation at 37°C. Quantification of 72 kDa MMP-2 activity measured by gelatin zymography. N = 4/group. * p<0.05 versus control (phosphorylated MMP-2 treated with 0 µM ONOO^−^).
(TIF)

## References

[1] Walsh CT, Garneau-Tsodikova S, Gatto GJ Jr (2005) Protein posttranslational modifications: The chemistry of proteome diversifications. Angew Chem Int Ed Engl 44: 7342–7372. 16267872 10.1002/anie.200501023

[2] Pontremoli S, Melloni E, Michetti M, Sparatore B, Salamino F, et al (1987) Phosphorylation and proteolytic modification of specific cytoskeletal proteins in human neutrophils stimulated by phorbol 12-myristate 13-acetate. Proc Natl Acad Sci USA 84: 3604–3608. 3473471 10.1073/pnas.84.11.3604 PMC304923

[3] Cadete VJ, Sawicka J, Jaswal JS, Lopaschuk GD, Schulz R, et al (2012) Ischemia/reperfusion-induced myosin light chain 1 phosphorylation increases its degradation by matrix metalloproteinase 2. FEBS J 279: 2444–2454. 22564771 10.1111/j.1742-4658.2012.08622.x PMC3377847

[4] Jiang ZG, McKnight CJ (2006) A phosphorylation-induced conformation change in dematin headpiece. Structure 14: 379–387. 16472756 10.1016/j.str.2005.11.007

[5] Groban ES, Narayanan A, Jacobson MP (2006) Conformational changes in protein loops and helices induced by post-translational phosphorylation. PLoS Comput Biol 2: e32. 16628247 10.1371/journal.pcbi.0020032 PMC1440919

[6] Volkman BF, Lipson D, Wemmer DE, Kern D (2001) Two-state allosteric behavior in a single-domain signaling protein. Science 291: 2429–2433. 11264542 10.1126/science.291.5512.2429

[7] Lee J, Giordano S, Zhang J (2012) Autophagy, mitochondria and oxidative stress: Cross-talk and redox signalling. Biochem J 441: 523–540. 22187934 10.1042/BJ20111451 PMC3258656

[8] Viappiani S, Schulz R (2006) Detection of specific nitrotyrosine-modified proteins as a marker of oxidative stress in cardiovascular disease. Am J Physiol Heart Circ Physiol 290: H2167–H2168. 16489112 10.1152/ajpheart.00128.2006

[9] Woessner JF Jr (1991) Matrix metalloproteinases and their inhibitors in connective tissue remodeling. FASEB J 5: 2145–2154. 1850705

[10] McCawley LJ, Matrisian LM (2001) Matrix metalloproteinases: They're not just for matrix anymore!. Curr Opin Cell Biol 13: 534–540. 11544020 10.1016/s0955-0674(00)00248-9

[11] Spinale FG (2007) Myocardial matrix remodeling and the matrix metalloproteinases: Influence on cardiac form and function. Physiol Rev 87: 1285–1342. 17928585 10.1152/physrev.00012.2007

[12] Schulz R (2007) Intracellular targets of matrix metalloproteinase-2 in cardiac disease: Rationale and therapeutic approaches. Annu Rev Pharmacol Toxicol 47: 211–242. 17129183 10.1146/annurev.pharmtox.47.120505.105230

[13] Kandasamy AD, Chow AK, Ali MA, Schulz R (2010) Matrix metalloproteinase-2 and myocardial oxidative stress injury: Beyond the matrix. Cardiovasc Res 85: 413–423. 19656780 10.1093/cvr/cvp268

[14] Wang W, Schulze CJ, Suarez-Pinzon WL, Dyck JR, Sawicki G, et al (2002) Intracellular action of matrix metalloproteinase-2 accounts for acute myocardial ischemia and reperfusion injury. Circulation 106: 1543–1549. 12234962 10.1161/01.cir.0000028818.33488.7b

[15] Ali MA, Chow AK, Kandasamy AD, Fan X, West LJ, et al (2012) Mechanisms of cytosolic targeting of matrix metalloproteinase-2. J Cell Physiol 227: 3397–3404. 22212960 10.1002/jcp.24040

[16] Cauwe B, Opdenakker G (2010) Intracellular substrate cleavage: a novel dimension in the biochemistry, biology and pathology of matrix metalloproteinases. Crit Rev Biochem Mol Biol 45: 351–423. 20812779 10.3109/10409238.2010.501783

[17] Beckman JS, Beckman TW, Chen J, Marshall PA, Freeman BA (1990) Apparent hydroxyl radical production by peroxynitrite: implications for endothelial injury from nitric oxide and superoxide. Proc Natl Acad Sci 87: 1620–1624. 2154753 10.1073/pnas.87.4.1620 PMC53527

[18] Viappiani S, Nicolescu AC, Holt A, Sawicki G, Crawford BD, et al (2009) Activation and modulation of 72 kDa matrix metalloproteinase-2 by peroxynitrite and glutathione. Biochem Pharmacol 77: 826–834. 19046943 10.1016/j.bcp.2008.11.004

[19] Sariahmetoglu M, Crawford BD, Leon H, Sawicka J, Li L, et al (2007) Regulation of matrix metalloproteinase-2 (MMP-2) activity by phosphorylation. FASEB J 21: 2486–2495. 17435175 10.1096/fj.06-7938com

[20] Sariahmetoglu M, Skrzypiec-Spring M, Youssef N, Jacob-Ferreira AL, Sawicka J, et al (2012) Phosphorylation status of matrix metalloproteinase 2 in myocardial ischaemia-reperfusion injury. Heart 98: 656–662. 22397940 10.1136/heartjnl-2011-301250

[21] Wang Z, Juttermann R, Soloway PD (2000) TIMP-2 is required for efficient activation of proMMP-2 in vivo. J Biol Chem 275: 26411–26415. 10827175 10.1074/jbc.M001270200 PMC2683068

[22] Villa LM, Salas E, Darley-Usmar VM, Radomski MW, Moncada S (1994) Peroxynitrite induces both vasodilatation and impaired vascular relaxation in the isolated perfused rat heart. Proc Natl Acad Sci USA 91: 12383–12387. 7809045 10.1073/pnas.91.26.12383 PMC45442

[23] Blom N, Gammeltoft S, Brunak S (1999) Sequence and structure-based prediction of eukaryotic protein phosphorylation sites. J Mol Biol 294: 1351–1362. 10600390 10.1006/jmbi.1999.3310

[24] Sreerama N, Woody RW (2000) Estimation of protein secondary structure from circular dichroism spectra: comparison of CONTIN, SELCON, and CDSSTR methods with an expanded reference set. Anal Biochem 287: 252–260. 11112271 10.1006/abio.2000.4880

[25] Laemmli UK (1970) Cleavage of structural proteins during the assembly of the head of bacteriophage T4. Nature 227: 680–685. 5432063 10.1038/227680a0

[26] Aureli L, Gioia M, Cerbara I, Monaco S, Fasciglione GF, et al (2008) Structural bases for substrate and inhibitor recognition by matrix metalloproteinases. Curr Med Chem 15: 2192–2222. 18781944 10.2174/092986708785747490

[27] Morgunova E, Tuuttila A, Bergmann U, Isupov M, Lindqvist Y, et al (1999) Structure of human pro-matrix metalloproteinase-2: activation mechanism revealed. Science 284: 1667–1670. 10356396 10.1126/science.284.5420.1667

[28] Okamoto T, Akaike T, Sawa T, Miyamoto Y, van der Vliet A, et al (2001) Activation of matrix metalloproteinases by peroxynitrite-induced protein S-glutathiolation via disulfide S-oxide formation. J Biol Chem 276: 29596–29602. 11395496 10.1074/jbc.M102417200

[29] Broncel M, Wagner SC, Paul K, Hackenberger CP, Koksch B (2010) Towards understanding secondary structure transitions: phosphorylation and metal coordination in model peptides. Org Biomol Chem 8: 2575–2579. 20485793 10.1039/c001458c

[30] Vepa S, Scribner WM, Natarajan V (1997) Activation of protein phosphorylation by oxidants in vascular endothelial cells: Identification of tyrosine phosphorylation of caveolin. Free Radic Biol Med 22: 25–35. 8958127 10.1016/s0891-5849(96)00241-9

[31] Rao GN (2000) Oxidant stress stimulates phosphorylation of eIF4E without an effect on global protein synthesis in smooth muscle cells. Lack of evidence for a role of H_2_0_2_ in angiotensin II-induced hypertrophy. J Biol Chem 275: 16993–16999. 10828072 10.1074/jbc.275.22.16993

[32] Wright VP, Reiser PJ, Clanton TL (2009) Redox modulation of global phosphatase activity and protein phosphorylation in intact skeletal muscle. J Physiol 587: 5767–5781. 19841000 10.1113/jphysiol.2009.178285 PMC2805384

[33] Radi R, Cosgrove TP, Beckman JS, Freeman BA (1993) Peroxynitrite-induced luminol chemiluminescence. Biochem J 290: 51–57. 8382481 10.1042/bj2900051 PMC1132381

