# Supplementary figures and images for: Phosphorylation Status of 72 kDa MMP-2 Determines Its Structure and Activity in Response to Peroxynitrite
Source: PLoS One. 2013 Aug 27;8(8):e71794. doi: 10.1371/journal.pone.0071794 (PMC3754950; doi:10.1371/journal.pone.0071794)

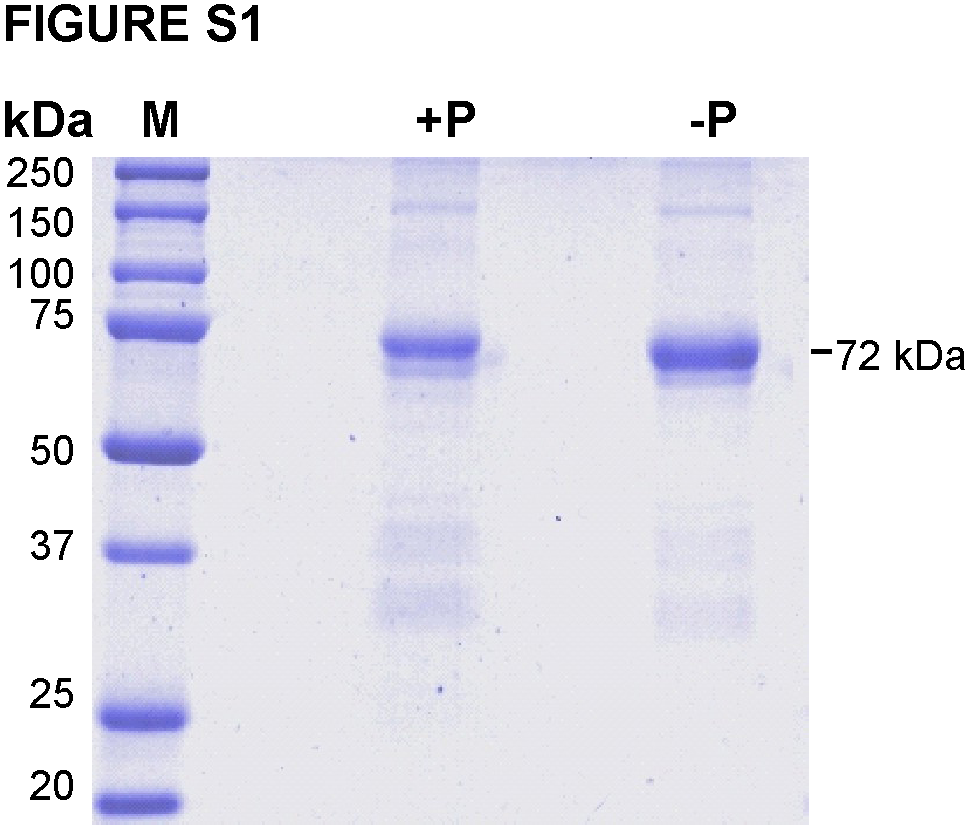

Supplement: Figure S1 — Purity of hrMMP-2. A) 10% Coomassie blue stained SDS-PAGE gel showing purified native (+P) or dephosphorylated (−P) hr 72 kDa MMP-2 used for CD studies. Expression and purification of hrMMP-2 were done as described in Methods. (TIF) [file pone.0071794.s001.tif]

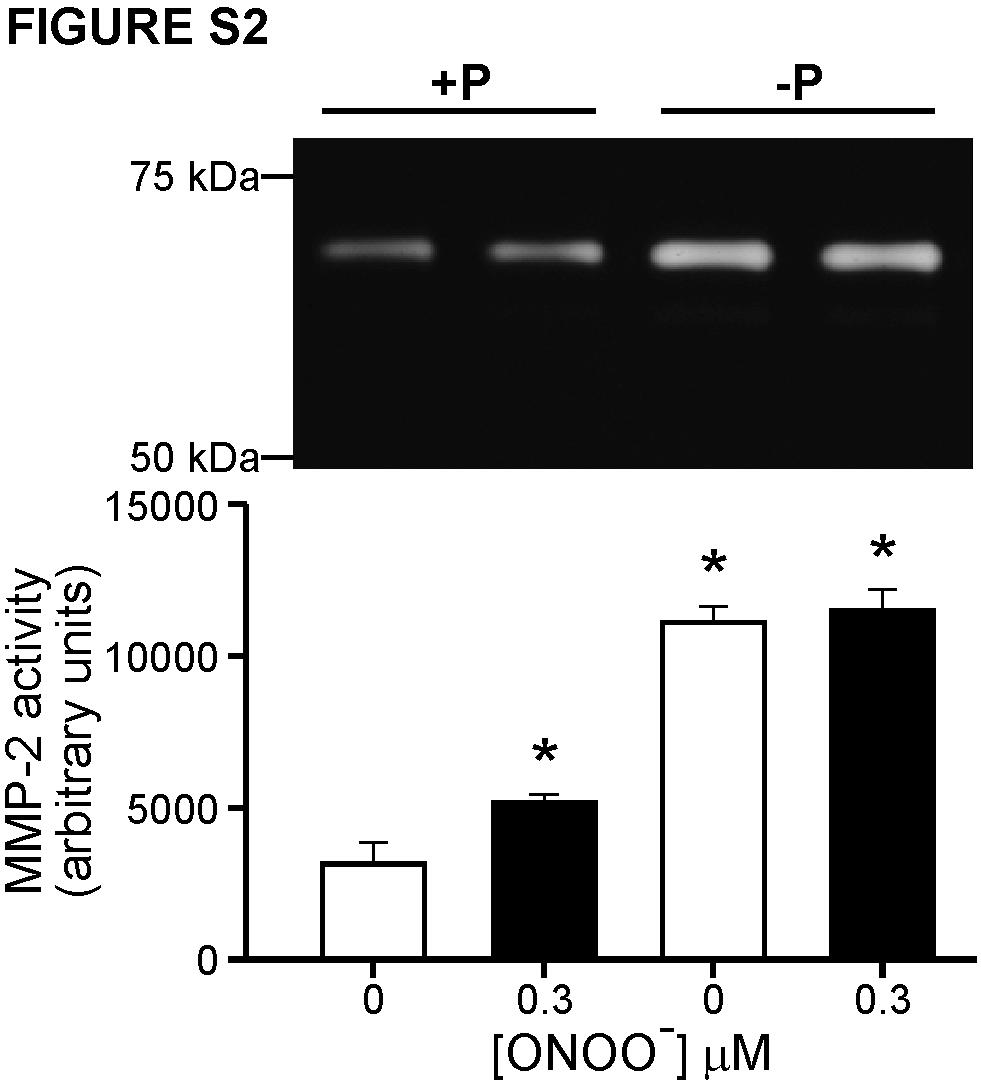

Supplement: Figure S2 — MMP-2 activity measured by gelatin zymography. Representative gelatin zymogram for 0–0.3 µM ONOO−-treated, native (+P) or dephosphorylated (−P) 72 kDa hrMMP-2, in the presence of 30 µM GSH, following 16 h of incubation at 37°C. Quantification of 72 kDa MMP-2 activity measured by gelatin zymography. N = 4/group. * p<0.05 versus control (phosphorylated MMP-2 treated with 0 µM ONOO−). (TIF) [file pone.0071794.s002.tif]
